# Successive harvests affect the aromatic and polyphenol profiles of novel catnip (*Nepeta cataria* L.) cultivars in a genotype-dependent manner

**DOI:** 10.3389/fpls.2023.1121582

**Published:** 2023-02-13

**Authors:** Erik Nunes Gomes, Harna Patel, Bo Yuan, Weiting Lyu, H. Rodolfo Juliani, Qingli Wu, James E. Simon

**Affiliations:** ^1^ New Use Agriculture and Natural Plant Products, Department of Plant Biology, Rutgers University, New Brunswick, NJ, United States; ^2^ Federal Agency for Support and Evaluation of Graduate Education (CAPES), Ministry of Education of Brazil, Brasilia, DF, Brazil; ^3^ Department of Medicinal Chemistry, Ernest Mario School of Pharmacy, Rutgers University, Piscataway, NJ, United States; ^4^ Center for Agricultural Food Ecosystems, Institute of Food, Nutrition & Health, Rutgers University, New Brunswick, NJ, United States

**Keywords:** β-caryophyllene, apigenin, caffeic acid, caryophyllene oxide, essential oil (EO), nepetalactone, rosmarinic acid

## Abstract

**Introduction:**

Catnip (*Nepeta cataria* L.) produces volatile iridoid terpenes, mainly nepetalactones, with strong repellent activity against species of arthropods with commercial and medical importance. Recently, new catnip cultivars CR3 and CR9 have been developed, both characterized by producing copious amounts of nepetalactones. Due to its perennial nature, multiple harvests can be obtained from this specialty crop and the effects of such practice on the phytochemical profile of the plants are not extensively studied.

**Methods:**

In this study we assessed the productivity of biomass, chemical composition of the essential oil and polyphenol accumulation of new catnip cultivars CR3 and CR9 and their hybrid, CR9×CR3, across four successive harvests. The essential oil was obtained by hydrodistillation and the chemical composition was obtained via gas chromatography-mass spectrometry (GC-MS). Individual polyphenols were quantified by Ultra-High-Performance Liquid Chromatography- diode-array detection (UHPLC-DAD).

**Results:**

Although the effects on biomass accumulation were independent of genotypes, the aromatic profile and the accumulation of polyphenols had a genotype-dependent response to successive harvests. While cultivar CR3 had its essential oil dominated by *E,Z*-nepetalactone in all four harvests, cultivar CR9 showed *Z,E*-nepetalactone as the main component of its aromatic profile during the 1^st^, 3^rd^ and 4^th^ harvests. At the second harvest, the essential oil of CR9 was mainly composed of caryophyllene oxide and (*E*)-β-caryophyllene. The same sesquiterpenes represented the majority of the essential oil of the hybrid CR9×CR3 at the 1^st^ and 2^nd^ successive harvests, while *Z,E*-nepetalactone was the main component at the 3^rd^ and 4^th^ harvests. For CR9 and CR9×CR3, rosmarinic acid and luteolin diglucuronide were at the highest contents at the 1^st^ and 2^nd^ harvest, while for CR3 the peak occurred at the 3^rd^ successive harvest.

**Discussion:**

The results emphasize that agronomic practices can significantly affect the accumulation of specialized metabolites in N. cataria and the genotype-specific interactions may indicate differential ecological adaptations of each cultivar. This is the first report on the effects of successive harvest on these novel catnip genotypes and highlights their potential for the supply of natural products for the pest control and other industries.

## Introduction

The fundamental processes of plant biology are controlled by a series of biochemical reactions collectively known as plant primary metabolism. Primary metabolites include sugars, amino acids, nucleotides and fatty acids and other compounds that are directly related to cellular homeostasis (Maeda, 2019). However, due to the complexity of ecological interactions, plants developed a more specialized metabolism focused on the production of a wide array of organic compounds, defined as secondary metabolites, that play a crucial role in processes such as tolerance to abiotic stresses, defense against herbivory and pathogens, attraction of pollinators and seed dispersal (Wink, 2018; Pott et al., 2019). Among the main products of plant secondary metabolism, volatile organic compounds (VOCs), metabolites with a low molecular weight and high vapor pressure at ambient temperatures, stand out by their importance in plant defense and reproduction (Picazo-Aragonés et al., 2020).

The largest group of plant VOCs are terpenes, lipophilic metabolites composed of one or more isoprene units (Gomes et al., 2021; Rosenkranz et al., 2021). Monoterpenes and sesquiterpenes are largely found as constituents of plant essential oils and have been extensively studied due to their biological implications (Tetali, 2019; Gomes et al., 2021). Due to their ecological functions, the structures of terpenes and other secondary metabolites are hypothesized to have been evolutionary molded to interfere with physiological processes of herbivores and microorganisms that competed with the plants for resources (Wink, 2018). Such features are fundamental for the current application of these compounds, since we learned how to explore this ecologically driven chemodiversity to improve our quality of life, applying plant essential oils, terpenes and other natural products in the formulation of perfumes, cosmetics, flavoring agents, antimicrobials, pesticides and repellents, among others.

The rising concern about the environmental and human health issues related to the use of synthetic chemicals as insecticides and insect repellents has increased the interest on the study of essential oils and related compounds as alternative methods of pest control (Nerio et al., 2010; Regnault-Roger et al., 2012; Chaaban et al., 2017). Among the most effective arthropod repellents of natural source are nepetalactones, volatile iridoid monoterpenes present in the essential oil of several *Nepeta* species (Lamiaceae), most notably *N. cataria* L., commonly known as catnip (Gomes et al., 2020a; Gomes et al., 2020c). Ethanolic extracts and essential oils from *N. cataria* as well as isolated nepetalactones have shown remarkable repellent effects against mosquitoes, bed bugs, ticks and other arthropods, with results comparable to the synthetic N,N-Diethyl-meta-toluamide (DEET), widely regarded as the gold standard of insect repellents (Reichert et al., 2019; Shi et al., 2021; González et al., 2022).

In addition to iridoid terpenes, catnip genotypes may also accumulate sesquiterpenes, mainly β-caryophyllene and caryophyllene oxide, as part of their aromatic profile (Gomes et al., 2020b). Those sesquiterpenes, although not as common as nepetalactones in arthropod repellent formulations, have been reported as potential natural ingredients for pest control strategies (Moraes et al., 2017; Plata-Rueda et al., 2018). In addition to bioactive aroma volatiles, *N. cataria* also produces a series of secondary metabolites characterized as polyphenols, such as apigenin, luteolin, caffeic acid and rosmarinic acid (Reichert et al., 2018), which have been reported as having direct influence on insect behavior (Onyilagha et al., 2012; Joshi et al., 2014; Khan et al., 2019) and can, therefore, be potentially used in integrated pest management systems. The study of those compounds, along with demonstrating sources of valuable natural products, can also provide insights into the function of secondary metabolites in plant ecophysiological interactions. The understanding of such interactions is important not only from an ecological perspective of understanding the evolutionary aspects of plant phytochemistry, but also from an agronomic perspective, as it can provide information on specific growing conditions to maximize the productivity of metabolites of industrial interest.

One of the main challenges associated with a steady supply of catnip secondary metabolites for industry is the scarcity of highly productive genotypes that offer a stable chemical profile and a consistent yield of aboveground biomass (Gomes et al., 2020a). Looking to address this issue, our team has recently developed two new catnip cultivars, CR3 and CR9, that show improved yields of biomass and essential oils rich in nepetalactones, and with superior productivity when compared to other commercially available catnip genotypes (Reichert et al., 2016; Simon et al., 2019). Although the chemical profile of most commercial catnip cultivars is also dominated by nepetalactones, both cultivars CR3 and CR9 are unique in their superior productivity due to higher essential oil contents and biomass productivity. Detailed comparisons of these new cultivars to other catnip genotypes in terms of essential oil productivity and composition are described by Reichert et al. (2016); Reichert et al. (2019) and Simon et al., 2019). In addition, Cultivars CR3 and CR9 were developed in part to have distinct chemical profiles, producing different nepetalactone isomers in their aromatic profile. While cultivar CR9 produces essential oils dominated by *Z,E*-nepetalactone, CR3 essential oil’s major compound is of *E,Z-*nepetalactone, followed by *Z,E*-nepetalactone (Reichert et al., 2016; Simon et al., 2019). Both cultivars show stability in terms of productivity of each of those isomers in similar cultivation conditions. However, the interactions of these newly developed genotypes with different agronomic practices have not yet been studied.

Due to their closely related evolutionary relationship, the productivity of secondary metabolites is strongly influenced by environmental conditions, including agronomic interventions (Gomes et al., 2021; Chrysargyris et al., 2022). Among the cultivation approaches that can affect the production of secondary metabolites in catnip, harvesting regimes have been reported to be of fundamental importance (Chalchat and Lamy, 1997; Mohammadi and Saharkhiz, 2011; Mohamed et al., 2018; Gomes et al., 2020b). Because catnip plants are perennial, successive harvests are a common practice for this aromatic crop and the effects of such practice on plant productivity and chemical profile have not been extensively studied. Although a few reports briefly discuss the effect of successive harvest on the terpene composition and biomass accumulation of some catnip genotypes (Mohamed et al., 2018; Gomes et al., 2020b), for the new cultivars CR3 and CR9 there have been no studies to date to determine the effects of this practice on the chemical profile in terms of both terpenes and polyphenols.

The present study aimed to characterize the aromatic profile and polyphenol accumulation of new catnip cultivars CR3 and CR9 and their hybrid (CR9×CR3) across four successive harvests as well as to assess if the effects of successive harvests on physiological and productive traits are genotype dependent. Moreover, this is the first description of the polyphenol composition of cultivars CR3 and CR9 as well as the first report on the chemical characterization of the polyphenol and aromatic profile of the self-pollinated progeny of their hybrid, CR9×CR3. The results are discussed in terms of ecophysiological interactions and potential applications of these highly productive genetic materials for the insect repellent industry and other markets.

## Materials and methods

### Genotypes, cultivation conditions and harvesting

The genetic materials used in the present study are recently patented catnip cultivars, named CR3 and CR9 and their previously uncharacterized hybrid, CR9×CR3. CR3 and CR9 produce *E,Z*-and *Z,E*-nepetalactones, respectively, as the major components of their essential oil and present higher biomass and essential oil production as well a more upright habit of growth than other commercially available genotypes (Reichert et al., 2016; Simon et al., 2019). CR3 and CR9 materials used in this study underwent two further generations of inbreeding under greenhouse conditions. Clones from these populations were then allowed to self-pollinate and the seeds resulting from this process were sowed to produce the seedlings used in the field study. To produce the CR9×CR3 hybrid, immature, unopened flowers from inbred CR9 plants were emasculated and, 24 hours later, hand pollinated with pollen from inbred CR3 plants. The pollinated inflorescences were covered with glassine bags (5x14 cm) until seed development. The seeds resulting from this process were then sowed and grown under greenhouse conditions in polypropylene pots (6.5 L) until full flowering, when the plants showing the highest growth in terms of biomass and height were selected and allowed to self-pollinate. The progeny obtained from this process underwent two generations of inbreeding and the clones were self-pollinated to produce seeds, as described to cultivars CR3 and CR9. Voucher specimens of the genotypes were deposited in the Chrysler Herbarium and Mycological Collection, Rutgers University (Catalog numbers: CHRB0021533, CHRB0021534 and CHRB0021535).

Propagation of the plants for the field experiments took place between April and May of 2017 under greenhouse conditions. Seeds of each of the 3 genotypes were sowed in semi-rigid, polypropylene, 128-cell plug trays (28 x 54cm) filled with commercial soil mixture (Canadian sphagnum peat moss, perlite, and an organic matter). The seeds were covered with a layer of about 0.1cm of commercial soil and gently watered once a day until soil saturation. A week after germination, seedlings were pricked out to ensure the growth of one seedling per cell in the tray. At 45 days after sowing, seedlings with 5 to 6 pairs of leaves and 15 to 20cm height (soil level to shoot apex) were selected to be transplanted into the field experiment.

The field experiments were carried out in an experimental farm in Pittstown, State of New Jersey, United States (40°33’26.7”N; 74°57’37.7”W;116m above sea level), with soil characterized as silt loam. The soil was prepared as described by Reichert et al. (2016), with base fertilization (N-P-K 15-15-15) applied to the soil at a rate of 1008.8 kg per hectare. Raised beds (0.9m wide) were mechanically established and covered with 0.32mm plastic mulch. A drip irrigation system was placed under the mulch and the plants were watered twice a week for 2 hours at an approximate rate of 3.4 liters per linear meter per hour.

The seedlings were transplanted in a single row on the raised beds in May of 2017. Eight seedlings of each genotype were transplanted into plots of 2.4m (by 0.9m wide) and spaced 0.3m apart from each other. Each plot of 8 plants was spaced 0.61m apart from each other. The raised beds were spaced 2.7m apart. Three plots of each genotype were established, and their positions were completely randomized inside each raised bed (blocks). Weed control was performed manually inside the raised beds and mechanically between raised beds. No intervention to control pests and diseases was necessary during the experiment. The monthly averages of weather conditions through the duration of the field experiment are presented in **Table 1**.

**Table 1:** Weather variables in Pittstown, NJ during the period of the experiment.

Year	Month	AcPrec	AvgRH	Avg SoilTemp 10cm	AvgTemp	MaxTemp	MinTemp	Avg SolarRad
		mm	%	°C				W/m²
2017	May	163.83	72.2	16.5	14.7	19.6	10.0	199.2
	June	74.168	67.5	21.4	20.9	26.2	15.2	258.4
	July	189.23	76.6	24.8	22.9	28.1	18.4	237.8
	Aug	91.948	77.2	23.4	20.9	26.0	16.3	215.4
	Sep	48.514	76.9	22.3	19.0	24.4	14.2	178.7
	Oct	109.22	75.2	19.6	15.3	20.5	10.2	129.6
	Nov	33.02	71.3	12.1	5.9	11.2	0.7	93.2
	Dec	36.83	70.4	7.2	-0.7	3.0	-4.3	67.8
2018	Jan	60.96	68.2	4.1	-2.7	1.9	-7.5	87.5
	Feb	155.956	78.2	7.6	3.1	7.8	-1.4	91.8
	Mar	106.426	66.5	5.7	2.0	6.2	-1.7	155.8
	Apr	120.396	64.1	10.1	7.7	13.1	2.7	179.2
	May	137.16	74.4	19.5	17.8	23.2	12.5	195.0
	June	60.96	70.8	25.7	20.5	26.0	15.2	252.4
	July	139.192	73.0	27.7	23.6	29.0	18.3	252.7
	Aug	210.82	82.3	27.3	23.3	28.3	19.2	211.2
	Sep	148.844	87.3	24.2	19.7	23.8	16.2	127.5

AcPrec, Accumulated Precipitation of the month; AvgRH, Average relatively humidity; AvgSoilTemp10cm, Average soil temperature at 10cm depth; AvgTemp, Average Temperature; MaxTemp, Average Maximum Temperature; MinTemp, Average minimum temperature; AvgSolarRad, Average solar radiation.

**Figure 1:**
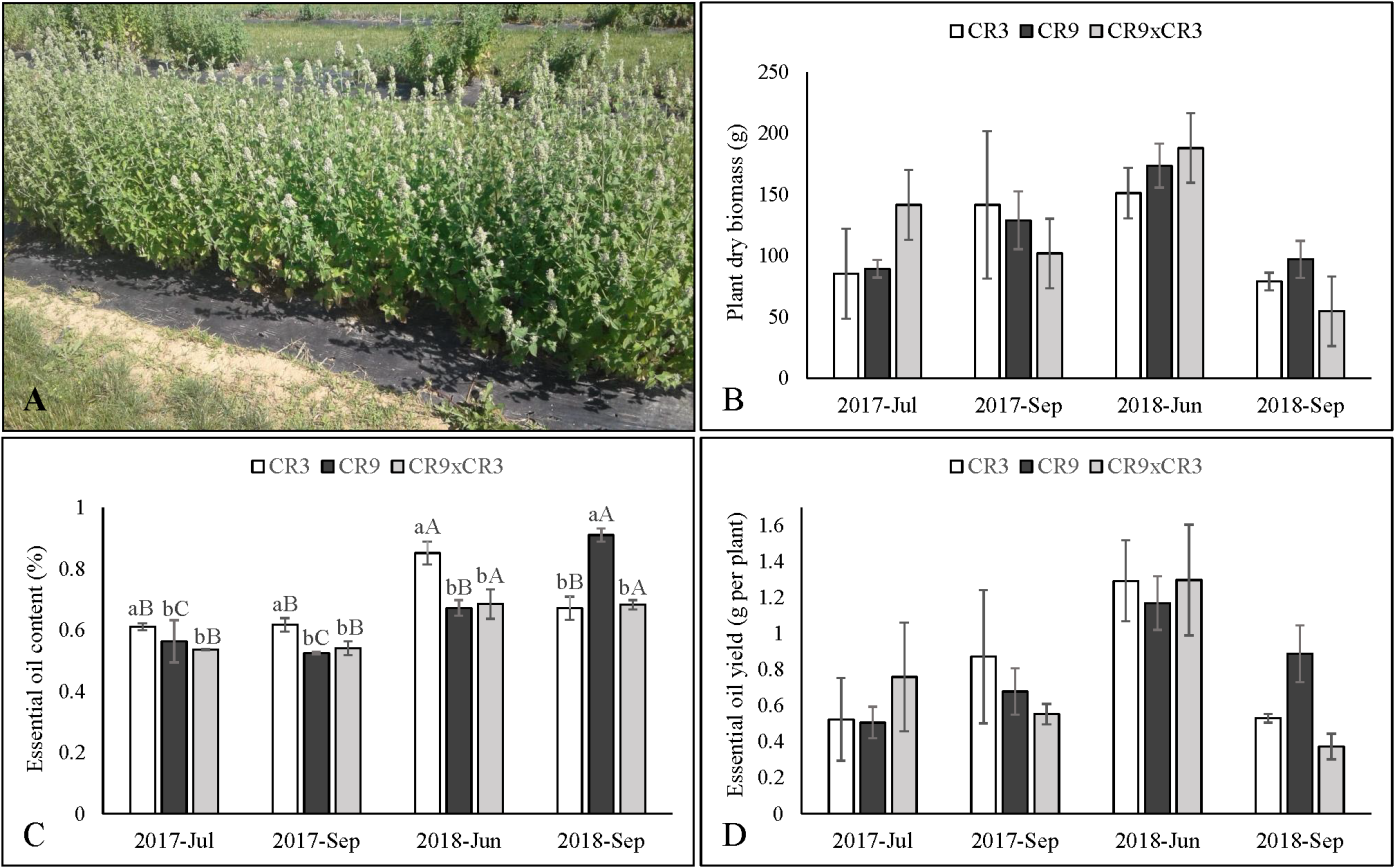
Aspect of catnip cultivar CR3 at harvest stage in September 2018 **(A)**, productivity of dry aboveground biomass **(B)**, essential oil content **(C)** and essential oil yield per plant **(D)** of catnip genotypes at different harvest times. Pittstown, NJ, United States. Vertical error bars indicate standard deviation. Uppercase letters indicate statistical comparisons of the same genotype across harvests and lowercase letters indicate comparisons among the three genotypes inside the same harvest. Averages with different letters are statistically different by the Scott-Knott test (*p ≤* 0.01). Plant Dry Biomass and essential oil yields do not contain letters because the interaction effects between genotypes and harvest times were not significant.

Harvest of the aboveground biomass of catnip was performed at the stage of full flowering, the period when the plants accumulate the highest amounts of compounds of interest, including nepetalactones (Chalchat and Lamy, 1997; Mohammadi and Saharkhiz, 2011; Ibrahim et al., 2017). The first harvest of the novel catnip genotypes took place 73 days after transplanting, in July of 2017. The plants were cut with hedge shears at approximately 10 cm above the soil level and the aboveground biomass was collected in brown paper bags (113.6 liters) for the determination of dry mass and phytochemical investigations. Weather permitting, *N*. *cataria* resumes its vegetative growth from the adventitious buds present in the lower stem nodes when harvested, which usually allows for two harvests per season and the maintenance of the plant stand for up to 3 years (Ferguson et al., 1990). Therefore, plants remained in the field and, 60 days after the first cut (September of 2017), reached the stage of full flowering, allowing for a second harvest in the year of 2017. Irrigation was interrupted once the plants were harvested in September 2017 and resumed in April 2018, after the plants overwintered in the field. Following overwintering, the new catnip genotypes presented a rate of survival of over 95% (data not shown), which allowed for the plants to be once again harvested, at full flowering, in June of 2018. Subsequent to the June 2018 cut, a second harvest of that growing season, forth harvest overall, was performed in September of 2018. **Figure 1A** shows a plot of catnip cv. CR3 at the stage of full flowering, after 3 successive harvests in September of 2018.

### Post-harvest handling, essential oil extraction and sample preparation

The harvested aboveground biomass (stems, leaves and inflorescences) of the catnip genotypes was placed into paper bags and dried at 37°C in a walk-in forced air tobacco dryer, as described by Reichert et al. (2016). The dried aboveground biomass of each plot was weighed, and the resulting mass was divided by 8 to determine biomass yield per plant.

The essential oil was extracted by hydrodistillation with a Clevenger-type apparatus (Clevenger, 1928). Briefly, 100g of dried ground biomass was added into 2L round bottom glass flasks and covered with 1 L of distilled water. The bottom of the flask was placed on an electric heating mantle and the top connected to a Clevenger trap coupled to a condenser. The mixture of water and plant material was boiled for 2 hours, and the condensed essential oil was collected from the Clevenger trap. The essential oil was weighed, and its content was determined as the proportion of the essential oil mass in relation to the mass of dried plant used for extraction (100 g), represented as a percentage (% of plant dry aboveground biomass). The essential oil distillation was performed in triplicate for each experimental unit and the average of the triplicate was used for statistical analysis. Essential oil yield was calculated by multiplying the essential oil content by the biomass yield per plant and was expressed in grams of oil per plant.

For essential oil composition analysis, an aliquot of 10 µL of essential oil was diluted in 1.5 mL of methyl tert-butyl ether (Chromatographic grade, Sigma Aldrich™). Approximately 1g of anhydrous Na_2_SO_4_ (Fisher Scientific™) was added to the solution. The mixture was centrifuged at 12000 rpm for 15 min and the supernatant was used for gas chromatography coupled to mass spectrometry (GC-MS) analysis.

For polyphenol analyses, samples were prepared from *N. cataria* dried leaves. After drying, leaves were manually separated from stems and inflorescences and were ground to a fine powder. Approximately 100mg of ground leaf powder were placed into 15ml polypropylene centrifuge tubes and diluted in 10ml of 70% methanol (ACS grade, Fisher Scientific™), 29.9% water (LC/MS grade, Fisher Scientific™), 0.1% formic acid (Optima™ LC/MS grade) solution. The mixture was sonicated for 15 minutes and stored at room temperature for 24 hours until chemical analysis. The material was then centrifuged at 12000 rpm for 10 min and the supernatant was used for the chemical analysis.

### Chemical analysis of essential oils by GC-MS

To determine the aromatic profile of catnip genotypes, 1 µL of essential oil diluted in methyl tert-butyl ether was injected into a Shimadzu 2010 Plus gas chromatograph supplied with an AOC-6000 auto-sampler. Helium (chromatographic grade, Airgas, Inc) was used as the carrier gas on a H-Rxi-5Sil MS column. The injection conditions and peak integration were performed as described by Reichert et al. (2019). To obtain the mass spectra of individual compounds, a Shimadzu TQ8040 triple-Q Mass Spectrometer was employed. The identification of the chemical constituents from the essential oils was performed by comparing their mass spectra to that in MS libraries (NIST05.lib, NIST05s.lib, W10N14.lib and W10N14R.lib) and further confirmation by comparison of calculated retention indices (from C8-20 n-alkane standard) to retention indices from the literature (Adams, 2007; Linstrom and Mallard, 2022). The identities of *Z,E*- and *E,Z*-nepetalactone, β-caryophyllene and caryophyllene oxide were further confirmed by comparison with authenticated standards. The chemical analysis was carried out in triplicate and the average was used in the statistical analysis. Yields of *Z,E*- and *E,Z*-nepetalactone, β-caryophyllene and caryophyllene oxide in mg per plant were estimated by multiplying their relative percentage in the essential oil by the productivity of essential oil per plant. We emphasize that these yields were meant as an approximation, based solely on the relative percentage of the peak areas and essential oil yield, with the objective to provide a combined analysis of the relative percentages of each compound and the essential oil productivity, and should not be used as a prediction of absolute yields of specific compounds.

### Chemical analysis of polyphenols *via* UHPLC-DAD

The method for polyphenol analysis was adapted from Reichert et al. (2018), with slight modifications. An Agilent 1290 Infinity II Ultra-High-Performance Liquid Chromatography (UHPLC) system coupled with The Agilent 6546 Quadrupole Time-of-Flight Mass Spectrometer (QTOF), featuring a dual Agilent Jet Stream (AJS) electrospray ionization (ESI) source and an Agilent 1290 Infinity II diode-array detection (DAD) Ultraviolet (UV) detector was used for analyzing the samples. The compounds were separated in a Waters Acquity UPLC BEH C18, 2.1 × 50 mm, 1.7μm column. The identification of phenolic acids and flavone aglycones were confirmed by authenticated standards and flavone glycosides were tentatively identified based on high-resolution mass spectrometry (HRMS). For data acquisition and analysis, the Agilent MassHunter Workstation Data Acquisition (version B.10.0) and Qualitative Analysis (version B.10.0) were used.

The mobile phase used for chromatographic separation of analytes consisted of 0.1% formic acid LC/MS grade in LC/MS grade water (solvent A) and LC/MS grade 0.1% formic acid in LC/MS grade acetonitrile (Fisher Scientific™) (solvent B). The elution gradient was 97% A and 3% B from 0 min to 6 min, 20% B from 6 min to 11 min and then 45% B from 11 min to 11.10 min. It was increased to 100% B from 11.10 min to 11.50 min. The column was washed for 1 min with mobile phase B and then equilibrated with 3% B prior to the next injection. After each injection, the needle was washed for 10s with 70% LC/MS grade methanol. The flow rate was set at 0.500 mL/min and the injection volume was 2μL. The total runtime for the method was 11.50 min with the eluent before 2.8 minutes and after 11 minutes routed to the waste. The column temperature was held constant at 30°C and the autosampler storage temperature was maintained at 4°C for the duration of the run. The diode-array detection was set to the wavelengths of 254nm, 320nm, 370nm, and 500nm with a spectrum scan range of 190nm to 400nm.

For the electrospray ionization parameters, high purity nitrogen gas, supplied by a Parker Balston Nitrogen NitroFlow 60NA™ nitrogen generator, was used as the drying, sheath and nebulizing gas. The nebulizer was set at 30psi while the drying gas and sheath gas were set at 250°C with a flow rate of 13L/min and 10L/min respectively. The capillary voltage was at 5.0 kV and the nozzle voltage was at 0.5 kV. The samples were analyzed in positive ion mode. Compounds were quantified *via* the UV detector at 320nm. Calibration curves for caffeic acid, rosmarinic acid, luteolin and apigenin were constructed using commercial standards from Sigma Aldrich™ to quantify the respective compounds. The standards for the calibration curve were prepared by doing a 2-fold serial dilution of caffeic acid (ca. 38.0 - 0.297 μg/mL), rosmarinic acid (ca. 25.35 – 0.198 μg/mL), luteolin and apigenin (ca. 19.85 μg/mL - 0.155 μg/mL). Luteolin and apigenin glucosides, glucuronides and diglucuronides quantification was estimated by using a correction factor of molecular weight ratio compared to their respective aglycones apigenin and luteolin (Reichert et al., 2018).

### Experimental design and statistical analysis

The field experiment was arranged as a split-plot scheme within a randomized complete block design, with the genotypes as plots and the harvests as subplots. The experiment was composed of 3 blocks and each experimental unit was composed of 8 plants. For polyphenol analysis, harvests in which a specific compound was not detected in all three blocks for one or more genotypes were excluded from the statistical analysis but are included in the same graph for visual comparison. The data was examined by an Analysis of variance (ANOVA) and, when significant, Scott-Knott *post-hoc* testing was applied. Statistical analyses were performed using the statistical software ASSISTAT 7.7 (Silva and Azevedo, 2016).

## Results

### Biomass and essential oil yields

Following ANOVA, the interaction effect between genotypes and harvest times was not significant for the accumulation of aboveground biomass (p=0.12) and essential oil yield per plant (p=0.08). The averages are presented in **Figures 1B**, **C** The main effect of genotype was not statistically significant for accumulation of biomass and essential oil yield (p=0.49 and 0.44, respectively), but harvest time was shown to influence those variables (p<0.0001). Plants harvested in June of 2018 accumulated more biomass (171.1g) than those harvested in July and September of 2017, which, in turn, presented a better performance than September 2018 (4^th^ successive harvest) (**Table 2**). Essential oil yield per plant was highest at the 3^rd^ successive harvest (1.25g) and the other harvests were not statistically different (**Table 2**).

**Table 2:** Main effect of genotype and harvest time on biomass yield and essential oil yield of novel catnip plants.

	Biomass yield	Essential oil yield
	(g per plant)	
Genotypes		
CR3	114.41^ns^	0.80 ^ns^
CR9	122.45	0.81
CR9xCR9	121.65	0.75
Harvest time		
July 2017	105.56 b	0.60 b
Sep 2017	124.31 b	0.70 b
June 2018	171.10 a	1.25 a
Sep 2018	77.05 c	0.60 b

Averages followed by different letters are statistically different by the Scott-Knott test (p=≤0.01). ns, not statistically different.

For essential oil content (% of aboveground biomass), a significant interaction effect was observed (p<0.0001), evidencing a genotype-specific response to harvest times. Cultivar CR3 reached its highest essential content (0.85%) at the 3^rd^ successive harvest (June 2018) and a lower value in all the other periods, while cultivar CR9 had the lowest contents in both 2017 harvests, followed by an increase in June of 2018 and reaching the highest value (0.91%) in the 4^th^ successive harvest, in September of 2018 (**Figure 1D**). The hybrid CR9×CR3 had its highest contents in both 2018 harvests (0.68%). Comparing genotypes, cultivar CR3 had the highest essential oil content in both harvests of 2017 and in June of 2018, while CR9 had the highest content amongst the genotypes in September 2018 (**Figure 1C**).

### Essential oil chemical composition

**Figure 2:**
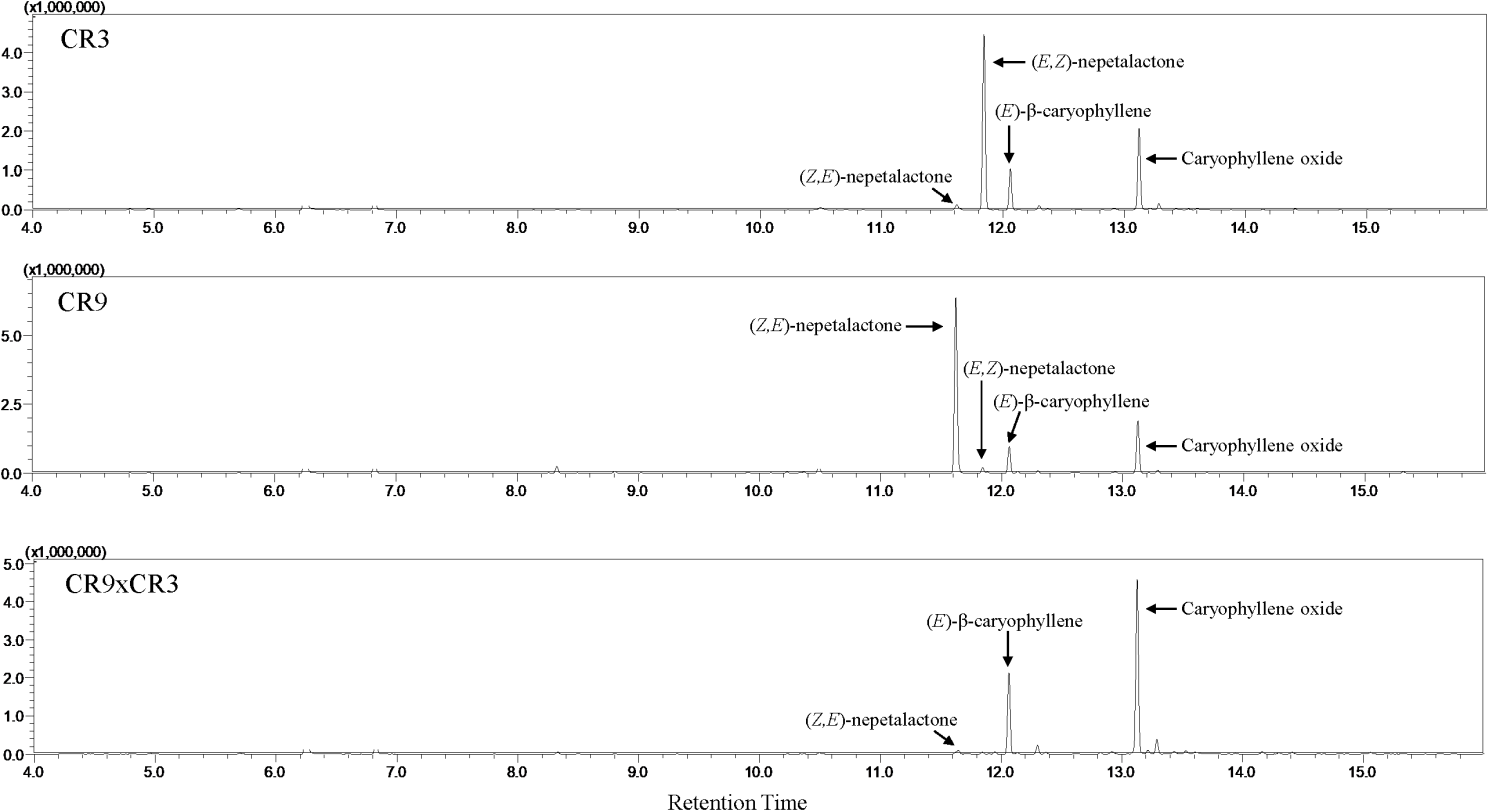
Representative GC chromatograms of the essential oils from catnip cultivars CR3 and CR9 and hybrid CR9×CR3 harvested in July 2017.

**Figure 3:**
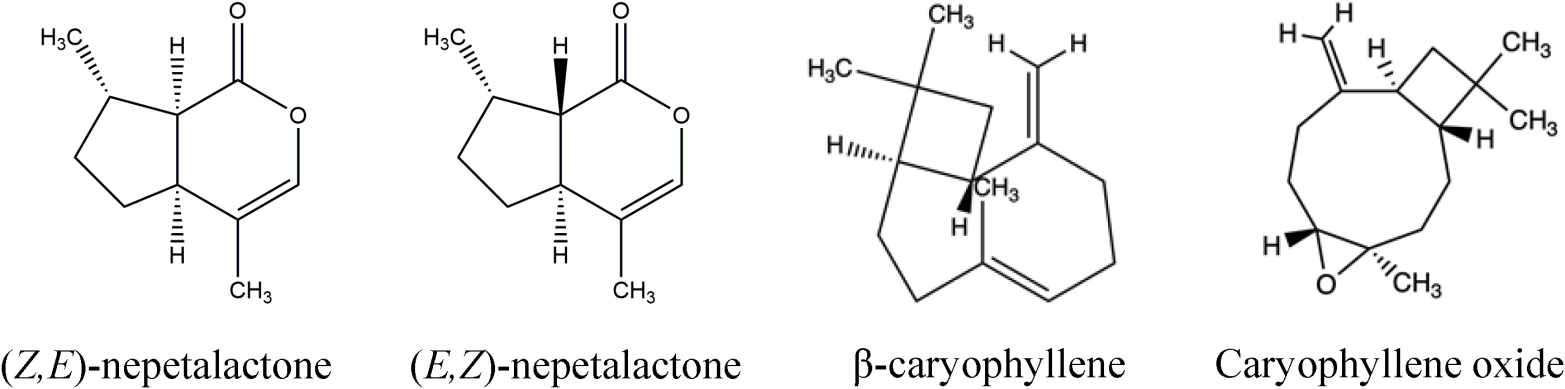
Chemical structures of *Z,E*-nepetalactone, *E,Z-*nepetalactone, β-caryophyllene and Caryophyllene oxide, the major components of the aromatic profile of catnip cultivars CR9, CR3 and hybrid CR9×CR3.

Twenty compounds were identified in the essential oils of the three new catnip genotypes across the four successive harvests (**Table 3**). **Figure 2** shows the representative chromatograms of the essential oils from cultivars CR3 and CR9 and hybrid CR9×CR3 harvested in July 2017, providing evidence of the presence of the 4 major compounds identified: *Z,E*-nepetalactone, *E,Z*-nepetalactone, (*E*)-β-caryophyllene and caryophyllene oxide. The chemical structures of the 4 major essential oil compounds identified in this study are presented in **Figure 3**.

**Table 3:** Chemical constituents and relative percentages of total chromatogram area of essential oils from different genotypes of catnip (*Nepeta cataria* L.) across 4 successive harvests in the years of 2017 and 2019. Pittstown, NJ, United States.

				June 2017			September 2017			June 2018			September 2018		
				*CR3*	*CR9*	*9x3*	*CR3*	*CR9*	*9x3*	*CR3*	*CR9*	*9x3*	*CR3*	*CR9*	*9x3*
RT	RI	RIL	Compound	*Area %*											
9.469	1097	1096	Linalool	–	–	–	T	–	–	T	–	–	–	–	–
9.932	1154	1158	Nerol oxide	–	–	–	T	–	–	T	–	–	–	–	–
10.228	1188	1184	Terpinen-4-ol	–	0.7 ± 0.2	–	T	T	–	T	0.7 ± 0.1	T	–	–	–
10.374	1204	1196	Myrtenal	T	–	–	T	0.9 ± 0.7	–	T	T	T	–	–	–
10.509	1224	1225	Citronellol	1.1 ± 0.5	3.0 ± 0.5	0.5 ± 0.5	3.5 ± 1.1	6.7 ± 2.1	6.4 ± 0.8	1.6 ± 0.7	3.1 ± 0.1	2.2 ± 0.8	0.9 ± 0.3	T	0.8 ± 0.2
10.628	1239	1238	Neral	–	–	–	T	–	–	–	–	T	T	–	–
10.696	1250	1252	Geraniol	T	–	–	T	–	–	T	–	T	T	–	–
10.842	1268	1267	Geranial	–	–	–	T	–	–	T	–	T	T	–	–
11.619	1372	1371	(*Z,E*)-nepetalactone*	3.5 ± 1.6	60.4 ± 10.7	1.3 ± 0.5	9.4 ± 5.6	18.2 ± 10.4	16.0 ± 4.8	28.3 ± 8.8	62.7 ± 3.7	69.5 ± 4.8	34.0 ± 12.2	89.8 ± 2.0	90.0 ± 1.6
11.843	1408	1405	(*E,Z*)-nepetalactone*	59.9 ± 11.3	1.8 ± 0.3	–	74.8 ± 16.1	1.1 ± 1.9	1.7 ± 0.6	55.4 ± 7.3	2.4 ± 0.4	5.7 ± 0.6	58.5 ± 11.5	0.9 ± 0.4	3.8 ± 0.7
11.945	1418	1415	(*Z*)-β-caryophyllene	–	T	T	–	–	T	–	T	T	–	T	–
12.059	1434	1433	(*E*)-β-caryophyllene*	10.7 ± 3.9	10.0 ± 2.8	28.5 ± 5.5	2.1 ± 2.1	17.5 ± 6.6	18.5 ± 4.3	5.0 ± 0.2	9.7 ± 0.7	8.0 ± 4.3	2.9 ± 0.8	1.2 ± 2.0	2.6 ± 0.1
12.131	1456	1457	(*E*)-β-farnesene	–	0.8 ± 0.2	–	–	–	–	–	0.7 ± 0.1	T	–	T	–
12.297	1469	1462	α-humulene	1.1 ± 0.4	1.0 ± 0.3	3.1 ± 0.6	T	1.4 ± 1.2	1.7 ± 0.4	T	0.9 ± 0.1	0.6 ± 0.4	T	T	T
12.367	1491	1488	(*E*)-β-ionone	T	–	0.8 ± 0.7	T	1.5 ± 0.1	1.5 ± 0.2	T	–	–	T	T	–
12.629	1532	–	Unidentified 1	T	–	T	T	1.5 ± 0.8	–	T	–	T	T	5.4 ± 1.5	T
13.121	1599	1596	Caryophyllene oxide*	21.3 ± 0.5	20.5 ± 6.4	57.2 ± 5.5	8.5 ± 7.0	43.2 ± 15.2	46.6 ± 0.4	8.2 ± 2.2	18.1 ± 3.0	11.9 ± 0.4	2.7 ± 0.4	1.6 ± 0.4	2.3 ± 0.6
13.211	1625	–	Unidentified 2	T	–	1.3 ± 0.2	–	–	–	–	–	–	–	–	–
13.284	1628	1620	Humulene epoxide II	1.6 ± 0.6	1.6 ± 0.4	5.0 ± 1	T	2.7 ± 1.1	2.8 ± 0.2	T	1.1 ± 0.2	0.6 ± 0.4	T	T	T
13.526	1667	1646	Aromadendrene epoxide	–	–	T	–	T	–	–	–	–	–	–	–
13.599	1688	1713	Pentadecanal	–	–	T	–	–	–	–	–	–	–	–	–
14.158	1797	–	Unidentified 3	–	–	–	–	T	1.0 ± 0.2	T	–	T	T	T	T
14.408	1840	1845	Hexahydrofarnesyl acetone	–	–	1.0 ± 0.1	T	4.2 ± 1,8	3.8 ± 1.6	–	–	–	T	–	–
15.615	2058	–	Unidentified 4	–	T	–	–	–	–	–	T	T	–	–	–
15.824	2096	–	Unidentified 5	–	–	–	–	–	–	–	–	T	T	–	–
**Total identified peaks**				**99.36**	**99.56**	**98.29**	**99.98**	**98.07**	**98.95**	**99.91**	**99.77**	**99.65**	**99.47**	**94.53**	**99.62**
Monoterpenes				64.52	65.90	1.80	89.41	27.33	24.10	85.94	69.21	78.03	93.81	91.07	94.63
Sesquiterpenes				34.84	33.66	95.16	10.36	70.54	71.05	13.97	30.56	21.62	5.62	3.46	4.99
Others				0.0	0.0	1.33	0.21	4.20	3.8	0.0	0.0	0.0	0.04	0.0	0.0

Data are the mean of three blocks ± Standard deviation. Compounds not detected in a sample were indicated as -. T: Trace amounts (lower than 0.5%). RT, Retention Time. RI, retention indices experimentally calculated using homologue series of n-alkanes (C8-C18); RIL, Retention indices from literature (Linstrom and Mallard, 2022); *Identity of compounds confirmed with authenticated standards; 9x3: CR9×CR3 hybrid of catnip cultivars CR9 and CR3.

Catnip cv. CR3 showed *E,Z*-nepetalactone as the major component of the essential oil in all 4 harvests, reaching a peak of 74.8% in September 2017. Catnip cv. CR9 had the isomer *Z,E*-nepetalactone as the main component of the essential oil in 3 out of the 4 harvests, with caryophyllene oxide being the major compound (43.2%) in September 2017. CR9xCR9 presented the essential oil dominated by sesquiterpenes, mainly caryophyllene oxide and (*E*)-β-caryophyllene, in both harvests of 2017, which coincides with the period when these genotypes showed the lowest essential oil contents. The hybrid had a similar aromatic profile to that observed in the parent genotype CR9 in the harvests of September 2017 and June and September 2018, including considerably high percentages of *Z,E*-nepetalactone (90%) at the 4^th^ successive harvest and caryophyllene oxide (46.6%) at the 2^nd^ successive harvest.

Total nepetalactone contents (sum of both isomers) in CR3 varied from 63.4% in the first harvest to 92.5% in the 4^th^ harvest, while for CR9 it varied from 19.3% in the 2^nd^ harvest to 90.7% in the 4^th^. CR9×CR3 varied from 1.3% in the first harvest to 93.8% in the 4^th^ harvest. The 4^th^ successive harvest shows the highest concentration of *Z,E-* and total nepetalactones in all genotypes (**Table 3**), which coincides with the only period when CR9 had a higher essential oil content than CR3 (**Figure 1C**) and the biomass yield was the lowest for the average of the genotypes (**Table 2**).

Apart from the insect repellent nepetalactones, the sesquiterpenes caryophyllene oxide and (*E*)-β-caryophyllene were significant components in the essential oils from the novel catnip genotypes, mainly CR9 and CR9×CR3 at the first two harvests. The relative contribution of sesquiterpenes linearly declined across harvests in CR9×CR3 (95.2%, 71.1%, 21.6% and 4.99% at 1^st^, 2^nd^, 3^rd^, and 4^th^ successive harvests, respectively). For CR9, a peak of sesquiterpenes (70.5%) was observed at the 2^nd^ successive harvest, followed by a marked decline in harvests 3 and 4 (30.6% and 3.46%, respectively). CR3 accumulated its highest percentage of sesquiterpenes at the first harvest followed by intermediate values at the 2^nd^ and 3^rd^ harvest and the lowest percentage at harvest 4, in September of 2018.

The sesquiterpenes α-humulene and humulene epoxide II, although not present in substantial volumes, were detected across all harvests in all genotypes. A similar pattern was observed for the monoterpene citronellol, varying from trace amounts to 6.7%. The highest percentages of citronellol were observed at the 2^nd^ successive harvest for all genotypes, time when the highest percentages of sesquiterpenes and lowest percentages of *Z,E-*nepetalactone were registered.

### Estimated yields of nepetalactones and sesquiterpenes

**Figure 4:**
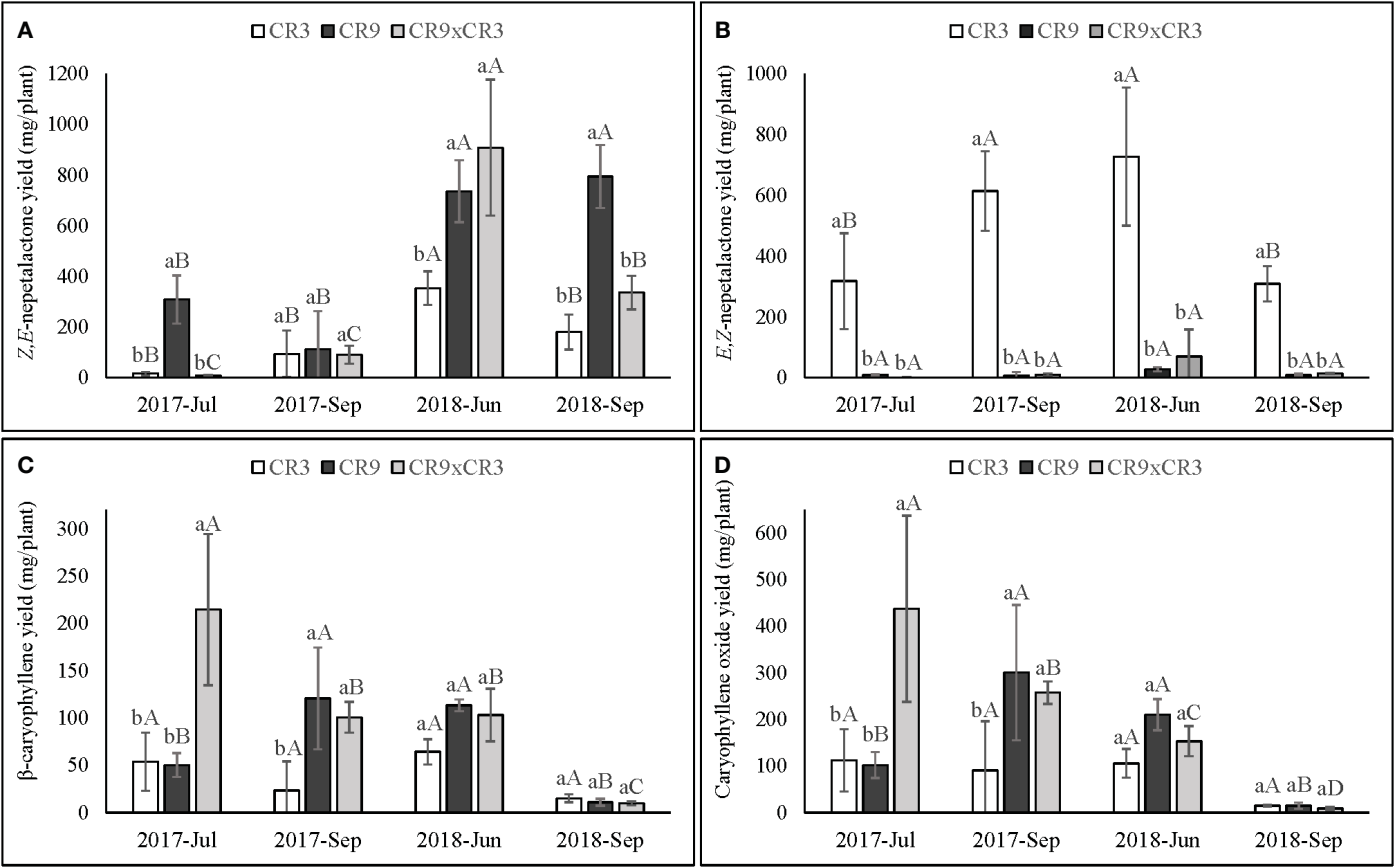
Yields (mg per plant) of *Z,E*-nepetalactone **(A)**, *E,Z-*nepetalactone **(B)**, β-caryophyllene **(C)** and Caryophyllene oxide **(D)** in catnip genotypes at different harvest times. Pittstown, NJ, United States. Vertical error bars indicate standard deviation. Uppercase letters indicate statistical comparisons of the same genotype across harvests and lowercase letters indicate comparisons among the three genotypes inside the same harvest. Averages with different letters are statistically different by the Scott-Knott test (*p*=≤0.01). Estimated as a function of relative percentage of the individual compounds in the essential oil and the essential oil yield.

The four major compounds (**Table 3**), *E,Z*- and *Z,E*-nepetalactone, caryophyllene oxide and (*E*)-β-caryophyllene were analyzed regarding their estimated yield per plant (**Figure 4**). These yields were calculated based on the relative percentage of the peak areas and the essential oil yield per plant and are meant as an estimate allowing for a combined analysis of the aromatic component and the total yield of essential oil produced. The yields of such compounds presented a similar pattern to that observed in their relative percentage peak area. Significant interaction effects between harvest times and genotypes were observed for the yields of all 4 compounds (p ≤ 0.01).

Cultivar CR9 showed yields of *Z,E*-nepetalactone in a pattern similar to that observed for essential oil content, with the highest values at the 3^rd^ and 4^th^ successive harvests (736 and 795mg per plant, respectively). CR9×CR3 reached a peak in June 2018 (908mg per plant), with an intermediate yield in September of 2018 (336mg) and the lowest values, up to 90mg per plant, for 1^st^ and 2^nd^ harvests. CR3 showed a peak of *Z,E*-nepetalactone yield in June of 2018 (354mg). CR9 had a superior *Z,E*-nepetalactone yield than those of both CR9×CR3 and CR3 at 1^st^ and 4^th^ successive harvest and was superior to CR3 also in the 3^rd^ successive harvest. The genotypes did not differ from each other in terms of *Z,E*-nepetalactone yield in September of 2017 (**Figure 4A**). *E,Z*-nepetalactone yield in CR3 was superior to CR9 and CR9×CR3 across all harvests. The highest yields of *E,Z*-nepetalactone in CR3 occurred at the 2^nd^ and 3^rd^ successive harvests (614 and 727mg per plant, respectively) (**Figure 4B**).

The yields of caryophyllene oxide and (*E*)-β-caryophyllene had a similar genotype-dependent response to successive harvests. CR9×CR3 hybrid showed a linear decrease on the yields of both sesquiterpenes, with the highest accumulation at the first harvest (214 and 437mg per plant, for (*E*)-β-caryophyllene and caryophyllene oxide, respectively), when the sesquiterpenes dominated the aromatic profile of this genotype. For CR9, the peak yields of both sesquiterpenes occurred at the 2^nd^ and 3^rd^ harvests, with a steep decline in the 4^th^ harvest. Cultivar CR3 did presented stable yields of sesquiterpenes, with no statistical differences across harvests for both caryophyllene oxide and (*E*)-β-caryophyllene (**Figures 4C, D**).

Accumulated biomass and essential oil yield (sum of harvests) for cultivar CR9 and the hybrid CR9×CR3 present similar values of accumulated biomass, around 490g in 2 years, slightly better than CR3, with 457.6g per plant. For essential oil yield, cultivars CR3 and CR9 had a similar performance, producing about 3.2g per plant after 4 harvests, and CR9×CR3 had a slightly smaller yield, around 2.98g per plant. CR9×CR3 showed the lowest variation between years for both biomass and essential oil yield (**Table 4**).

**Table 4:** Accumulated biomass and essential oil in novel catnip genotypes cultivated in 2017 and 2018. Pittstown, NJ, United States.

		Genotype		
		CR3	CR9	9X3
*g/plant*	Shoot Dry Biomass 2017	227.1	218.8	243.8
	Shoot Dry Biomass 2018	230.6	271.0	242.9
	Shoot Dry Biomass 2017 and 2018	457.6	489.8	486.6
*g/plant*	Total Essential oil yield 2017	1.39	1.18	1.31
	Total Essetial oil Yield 2018	1.82	2.05	1.67
	Total Essential oil Yield 2017 and 2018	3.21	3.24	2.98

### Polyphenol contents

**Figure 5:**
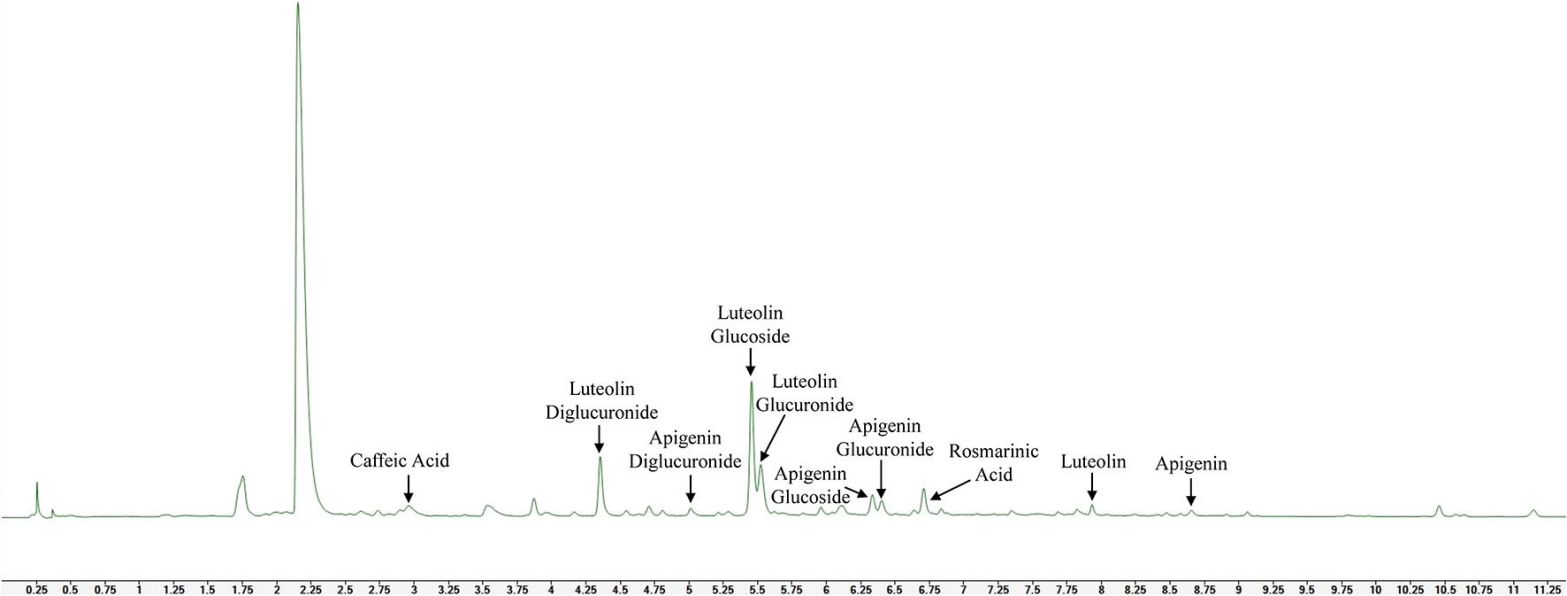
Representative UHPLC/UV chromatogram (320 nm) evidencing phenolic acids, flavones and flavone glycosides in methanolic extracts of leaves from catnip cultivar CR3.

Phenolic acids caffeic acid, rosmarinic acid and flavones luteolin, apigenin and their glucosides, glucuronides and diglucuronides were identified in the leaves of novel catnip genotypes in the present study, as shown in the representative UHPLC/UV chromatogram of the polyphenol profile of cultivar CR3 harvested in July of 2017 (**Figure 5**).

**Figure 6:**
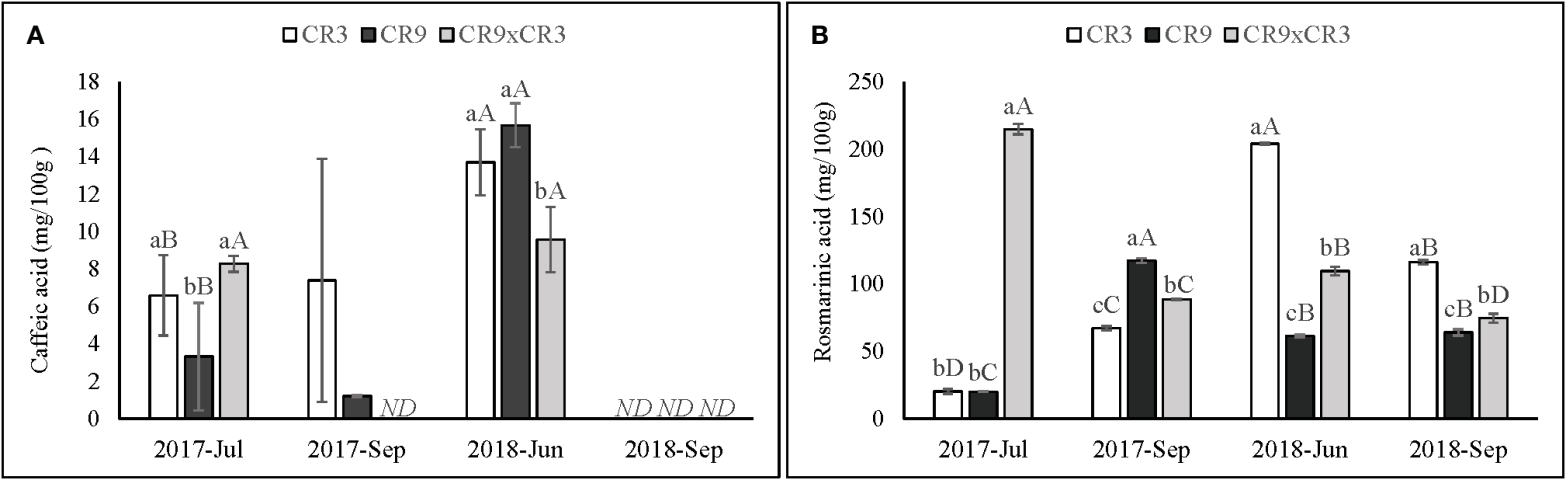
Content of caffeic acid **(A)** and rosmarinic acid **(B)** in catnip genotypes at different harvest times. Pittstown, NJ, United States Vertical error bars indicate standard deviation. Uppercase letters indicate statistical comparisons of the same genotype across harvests and lowercase letters indicate comparisons among the three genotypes inside the same harvest. Averages with different letters are statistically different by the Scott-Knott test (*p*=≤0.01). *ND*, not detected. Because caffeic acid was not detected in any of the genotypes for plants harvested in September 2018 and in genotype CR9×CR3 for plants harvested in September 2017, those harvests have not been included in the statistical analysis.

A significant (p ≤ 0.01) interaction effect between harvest and genotypes was observed for all phenolic compounds identified in this study, except for apigenin, which was only detected in the first harvest. Caffeic acid was not detected in any genotypes in September of 2018 and in CR9×CR3 harvested in September 2017. Additionally, in September 2017, it was found only in small amounts in cultivar CR9. For the harvests when caffeic acid was present in significant quantities, the accumulation showed a genotype-dependent effect (p=0.026). Both cultivars CR3 and CR9 showed higher contents in June 2018 than in July 2017, while CR9×CR3 showed statistically equal contents in both harvests (**Figure 6**).

CR9×CR3 showed the highest rosmarinic acid content amongst the genotypes in July 2017 (214mg/100g), followed by a decrease in subsequent harvests, reaching its lowest at the 4^th^ successive harvest (74 mg/100 g), with a pattern similar to that observed for its accumulation of sesquiterpenes. CR9 reached its peak accumulation of rosmarinic acid in September 2017 (117 mg/100 g), where it was also characterized by the high relative amounts of sesquiterpenes in the essential oil. Cultivar CR3 reached its highest contents of rosmarinic acid in 2018, with a peak in the first harvest of that season (204 mg/100 g) (**Figure 6**).

**Figure 7:**
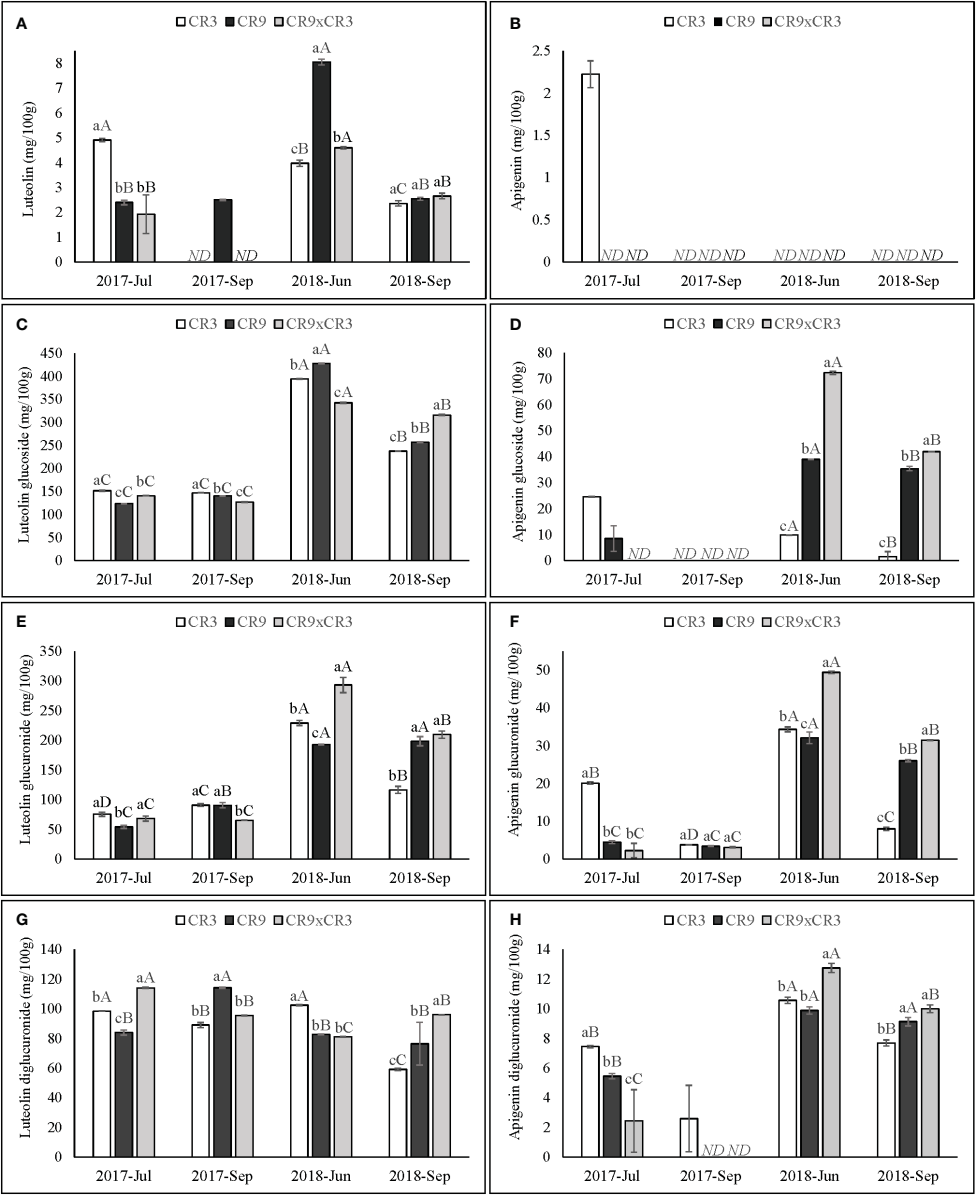
Contents of luteolin **(A)**, apigenin **(B)**, luteolin glucoside **(C)**, apigenin glucoside **(D)**, luteolin glucuronide **(E)**, apigenin glucuronide **(F)**, luteolin diglucuronide **(G)** and apigenin diglucuronide **(H)** in catnip genotypes at different harvest times. Pittstown, NJ, United States. Vertical error bars indicate standard deviation. Uppercase letters indicate statistical comparisons of the same genotype across harvests and lowercase letters indicate comparisons among the three genotypes inside the same harvest. Averages with different letters are statistically different by the Scott-Knott test (p=≤0.01). ND, not detected. Harvests in which a specific compound was not detected in one or more genotypes were excluded from the statistical analysis of that compound.

CR3 had its peak luteolin content (4.9 mg/100 g) at the first harvest of 2017. That harvest was also characterized by being the only period in this study when apigenin was detected (2.2 mg/100 g in cultivar CR3). Both cultivars CR9 and the hybrid CR9×CR3 reached their highest accumulation of luteolin at the 3^rd^ successive harvest (June 2018) and the lowest contents in both harvests of 2017. A similar pattern was observed for all genotypes for contents of luteolin and apigenin glucosides and glucuronides and apigenin diglucuronide. For luteolin diglucuronide, CR3 exhibited highest concentrations at the 1^st^ and 3^rd^ harvests (98 and 102 mg/100 g, respectively), while CR9 reached a peak at the 2^nd^ harvest (114mg/100g) and CR9×CR3 at the first harvest (114 mg/100 g). (**Figures 7A–H**).

At both harvests of 2018 (3^rd^ and 4^th^ successive harvests), hybrid CR3xCR9 was statistically higher to CR3 and CR9 on the accumulation of apigenin glucoside, glucuronide and diglucuronide and, at the 4^th^ successive harvest, for luteolin glucoside. Catnip cv. CR9 performed the best among genotypes at the 3^rd^ successive harvest for luteolin and luteolin glucoside contents. Catnip cv. CR3 had the highest contents of luteolin and luteolin glucoside, apigenin and apigenin glucoside, glucuronide and diglucuronide at the amongst the genotypes at the first harvest (**Figure 7**).

## Discussion

In this study, we demonstrated a strong genotype-dependent response to successive harvests on the accumulation of secondary metabolites in novel catnip genotypes. However, for the productivity of aboveground biomass and total essential oil yield per plant (essential oil content multiplied by aboveground biomass), the interaction was not significant and only the main effect of harvest significantly affected those variables. Biomass accumulation appears to be determined by primary metabolism and the genes involved in those pathways are known to be more conserved, highly regulated, and connected in more complex gene networks when compared to genes involved in secondar metabolism pathways (Maeda, 2019; Moore et al., 2019). Results suggest that primary metabolite-related genes are, overall, less susceptible to drastic variations caused by environmental interactions. Additionally, *N. cataria* cvs. CR9 and CR3 were specifically developed through selective breeding to be highly efficient in terms of biomass accumulation, with productivities superior to other available genotypes (Reichert et al., 2016; Simon et al., 2019). Given the superior performance of those cultivars, we hypothesize that the lack of significant differences among genotypes for biomass accumulation in this study is due to species-related physiological limitations to further increases. Additional evidence to support this possibility is the observation that the hybrid CR3xCR9 seems to produce similar, but not surpass, the parent plant’s biomass accumulation, demonstrating that a limit to further biomass increase could be added by factors not yet addressed in current catnip breeding programs.

Despite significant differences on the aromatic profile and polyphenol contents, results from the accumulated yield over 4 harvests (**Table 4**) show that the productive performance of these novel catnip genotypes is highly similar, with yields of about 500g of dried aboveground biomass and 3g of essential oil per plant. If multiplied by the plant density recommended by Park et al. (2007) for North American conditions, 80,742 plants per hectare, these new genotypes would yield about 242 kg of essential oil in 4 harvests, or about 60.5 kg per harvest per hectare. Those extrapolations are to be considered cautiously, as a high density of plants will likely reduce the individual productivity per plant, but they can be used to highlight the productive potential of these genetic materials. Such yield extrapolations are often higher than what growers often experience in commercial settings with larger field plots in which less than ideal conditions are the norm and where actual efficiencies in handling, harvesting and processing is less than what can often be achieved with small experimental plots.

As for the main effect of harvest on biomass and essential oil yield, it is possible to observe that the peak productivity occurs at the 3^rd^ successive harvest, first harvest after overwintering, after one year of establishment in the field and with the longest interval in between cuts. This emphasizes the importance of developing genotypes capable of surviving winter temperatures in North America since the peak productivity of biomass and essential oil occurs after one year in the field. Future studies should address possible harvest regimes to maximize production, such as not harvesting twice in the first year or, possibly, not harvesting at all, thinking on establishing a more productive stand for subsequent years. Similar approaches have been studied for other specialty crops such as stevia (Bihan et al., 2021) and blueberries (Strik and Buller, 2005).

For the essential oil content and composition as well as polyphenols, a clear genotype-dependent effect was observed, with contrasting responses mainly when comparing cultivars CR3 and CR9. The essential oil content in cultivar CR9 was the highest at the 4^th^ successive harvest, not following the pattern of decline observed for its biomass accumulation. For the hybrid CR9×CR3, the highest essential oil yield was observed in both 2018 harvests, also not following trends of biomass accumulation, which presented a steep decline in the second harvest of 2018 (4^th^ successive harvest). Such behavior illustrates the differences between responses of primary and secondary metabolism and the fact that secondary metabolites are more likely to vary according to cultivation practices and environmental conditions. It is possible to infer that the exposure to field conditions, including temperature stress, herbivory and the successive harvests hindered the ability of cultivar CR9 and hybrid CR9×CR3 to accumulate biomass after the 3^rd^ successive harvest and the highest accumulation of essential oils, terpenes and phenolics at that time may indicate their role as stress mediators. Accumulation of stress induced metabolites such as terpenes and phenolic compounds has been reported for other aromatic species subjected to successive harvests, such as basil (*Ocimum basilicum* L.), salad burnet (*Sanguisorba minor* Scop) and mints (*Mentha arvensis* L. and *M. × piperita* L.) (Ceccanti et al., 2019; Ciriello et al., 2021; Soltanbeigi et al., 2021). Although we did not directly assess herbivore incidence and abiotic stress, it is reasonable to infer that the longer the plants stay under the field conditions, the more opportunities for there to be exposure to biotic and abiotic stresses. In addition to direct herbivore interactions, one can also consider that the effect of cutting the plants during harvest can be in itself a stress perceived by the plant as similar to herbivory. In cotton plants (*Gossypium hirsutum* L.), for example, the accumulation of terpenes caused during herbivore-plant interactions has been shown to be a general wound response, which can be induced by mechanical wounding alone (Opitz et al., 2008). Previous research has also indicated that chemical defenses can be elicited in different plants species simply by the vibrations caused by insect feeding (Appel and Cocroft, 2014). Therefore, the accumulated effects of the two previous harvest can also be a strong contributor to the metabolite accumulation observed in harvests 3 and 4. Previous research on catnip have indicated that environmental stresses causing reduction on plant growth have also promoted increases on the contents of essential oil (Said-Al Ahl et al., 2016; Mohammadi et al., 2017), corroborating the results of the present study. The distinct response of cultivar CR3 further emphasizes the genotype-dependent nature of environmental interactions in catnip.

The contrast between the responses of cultivars CR3 and CR9 is also evident when considering the relative percentages of nepetalactones in the essential oil (**Table 3**) and the estimated yield of *Z,E*- and *E,Z*-nepetalactone (**Figure 4**). CR3 shows relatively stable chemical profiles across all harvests, with *E,Z*-nepetalactone dominating the essential oil. The changes on the yield of *E,Z*-nepetalactone per plant are, therefore, an expression of the trends of biomass accumulation and also reflect that CR3 produces more of that isomer than cultivar CR9 and CR9×CR3 hybrid. For cultivar CR9 and the hybrid CR9×CR3, the peak essential oil contents coincide with the harvests when *Z,E-*nepetalactone was present at the highest relative contents and, to some extent, also coincide with the highest yields of *Z,E*-nepetalactone (mg per plant). The hybrid CR9×CR3 has an aromatic profile more similar to that of CR9 than CR3. The enzymes involved in the production of different stereoisomers of nepetalactone have been recently identified as nepetalactol-related short-chain dehydrogenase enzymes (NEPS1, NEPS2 and NEPS3) (Lichman et al., 2019) and the differences observed among genotypes in the present study may be related to differential regulation of the expression and activity of those enzymes by environmental conditions. Greater knowledge about those enzymes will aid in breeding programs to explore the selection of more productive biosynthetic pathways of nepetalactones.

The biosynthesis and accumulation of nepetalactones in *Nepeta* species is known to take place in glandular trichomes on the leaf surface (Aničić et al., 2018) and changes on the amount (density) and morphology of such structures is known to correlate with the production of secondary metabolites in aromatic plants (Deschamps et al., 2006; Souza et al., 2016). Previous research has identified that glandular trichomes of different chemotypes of *N. cataria* differ in their structure and morphology (Kolalite, 1998), therefore, it is possible that part of the chemical contrast observed between cultivars CR3 and CR9 are correlated to differences in their glandular trichomes, a topic worthy of further research. Additionally, the density of glandular trichomes on the surface of *N. cataria* leaves is also known to be affected by environmental conditions such as saline stress (Lungoci et al., 2022) and fertilization (Paria et al., 2021). The investigation of trichome density and morphology in *N. cataria* and their correlation with agricultural practices and genotypes can be of fundamental importance for breeding programs as this is a trait directly associated with the productivity of compounds of interest in the species.

An interesting trend observed in the essential oil of CR9×CR3 was that in both harvests of 2017, sesquiterpenes, mainly caryophyllene oxide and (*E*)-β-caryophyllene, dominated the essential oil and, when the levels of those compounds were reduced (3^rd^ and 4^th^ successive harvests), *Z,E-*nepetalactone levels were high. A similar trend was observed for CR9, with the exception of the first harvest. Because sesquiterpenes are known to be involved in the regulation of lateral roots development and plant-herbivore interactions (Ditengou et al., 2015), one could infer that the synthesis of such compounds can have ecological advantages during the establishment of the plants in the field and, after which, the characteristic nepetalactone metabolic pathway can be fully expressed. The finding that CR3 presented smaller variation and smaller percentages of sesquiterpenes in its essential oil may indicate that this cultivar has a different root system, which allows it to establish faster than the other genotypes.

Previous studies have shown that some catnip genotypes can produce caryophyllene oxide and (*E*)-β-caryophyllene among the main components of their aromatic profiles (Gomes et al., 2020b; Said-Al Ahl et al., 2018). In addition to being involved with plant-environment interactions, such compounds have also been identified as potential tools for the use as natural repellents and/or insecticides (Moraes et al., 2017; Plata-Rueda et al., 2018) and can be included in integrated pest management systems including catnip extracts and nepetalactones. Furthermore, the constant presence of citronellol in all the studied genotypes, although not in high relative percentages, adds an active ingredient to the blend of terpenes that compose the essential oil of catnip, since this compound has been extensively reported to be a potent natural arthropod repellent (Mota et al., 2022). The essential oil blend containing several active ingredients for arthropod repellency, with different molecular weighs and biosynthetic pathways, and, possibly different modes of action, reinforces that the products from these new catnip genotypes can be part of an efficient strategy to slow down the development of resistance on pest populations and potentially to reduce the use of synthetic repellents.

A similar trend to that observed for sesquiterpenes occurred with the accumulation rosmarinic acid in CR9 and CR9×CR3, with higher accumulations when the levels of *Z,E*-nepetalactone were lower. Rosmarinic acid has been extensively described as a chemical component of catnip leaves and have been reported as the main responsible for the antioxidant properties of different extracts from this species (Dienaitė et al., 2018; Sharma et al., 2019). In addition to medical uses, rosmarinic acid has been identified as a potent biopesticide against aphids of agricultural importance (Khan et al., 2019), which further establishes the new catnip genotypes addressed in this study as sources of multiple phytochemical ingredients for use in pest control. Ecophysiologically, rosmarinic acid is known to be a mediator of plant environmental interactions and is recognized to be accumulated under biotic and abiotic stresses (Dewanjee et al., 2014; Mousavi and Shabani, 2019), which, in the context of CR9 and CR9×CR3 is possibly related to the period of adaptation to field conditions.

A comparable pattern to that of rosmarinic acid can be observed, although not as evident, is the accumulation of luteolin diglucuronide in CR9 and CR9×CR3, with peaks when those genotypes presented the highest concentrations of sesquiterpenes in their essential oils. This compound is not as frequently studied as other phenolic acids and flavones described in this study, but has been studied along with apigenin diglucuronide, regarding their anti-inflammatory and antinociceptive properties (Bueyuekokuroglu et al., 2008).

Similar to rosmarinic acid, caffeic acid has also been reported as an insecticide against pests of agricultural importance (Joshi et al., 2014) and is known to be involved in plant-herbivore interactions (Kundu et al., 2018). In the present study, for all genotypes, caffeic acid was found in higher amounts on the first harvests of each season. Those periods correspond to the highest values of solar radiation in the months when the plants have been harvested (July of 2017 and June of 2018) (**Table 1**). Such information highlights the possible involvement of caffeic acid in catnip responses to radiation stress, as this compound has been previously described to stabilize the absorption of high energy radiations in mesophyll cells, preventing oxidative stress (Riaz et al., 2019).

Despite some minor variations regarding the specificities of each genotype, a general tendency of higher accumulation of caffeic acid, luteolin, luteolin glucoside, luteolin glucuronide, apigenin glucoside, apigenin glucuronide and apigenin diglucuronide were observed to occur in the first harvest of 2018 (3^rd^ successive harvest). In addition to the previously mentioned radiation-induced biosynthesis, the 3^rd^ successive harvest was also the first harvest after the plants overwintered in the field and the presence of these compounds may be a residual effect of the protection mechanisms put in place to protect those genotypes from oxidative damages caused by cold stress. Each of these phenolic compounds and their glycosides have been previously reported to be involved in antioxidant metabolism (Hojati et al., 2011; Šliumpaitė et al., 2013; Lee et al., 2015; Bian et al., 2017; Vukić et al., 2022). Another perspective is to understand consider such compounds as indicators of more recent stress, since after their peak, the plants presented a steep decline in biomass accumulation. Many of the phenolic compounds identified in the novel catnip genotypes have been reported to inhibit herbivory (Erhard et al., 2007; Cipollini et al., 2008; Goławska and Łukasik, 2012; Joshi et al., 2014), which may indicate that the plants were employing defense mechanisms to avoid herbivory, and possibly, as a response to the successive harvests. Further studies on the involvement of phenolic compounds and catnip primary and secondary metabolisms are of strategic importance, since those compounds can be used as breeding traits for stress resistance, for use in pest control programs or as indicators of the crop’s physiological status.

This is the first description of the polyphenol composition of the new catnip cultivars CR9 and CR3 and the first description of the polyphenols and aromatic profile of the hybrid CR9×CR3. The genotype-dependent responses to successive harvests emphasize the need for future studies to understand how these new, highly productive, genotypes interact with the environment to maximize the productivity of compounds of interest.

## Conclusion

Biomass accumulation and essential oil yield (grams per plant) in new catnip cultivars CR3 and CR9 and their hybrid CR9x CR3 do not vary significantly as a function of genotype, but are strongly influenced by successive harvests, reaching a peak at the 3^rd^ successive harvest (first harvest after overwintering in the field) and with a decline at the 4^th^ successive harvest. In contrast, the chemical composition of the essential oil as well as the polyphenol accumulation present clear genotype-dependent responses to successive harvests. While cultivar CR3 shows *E,Z*-nepetalactone as the major component of its essential oil in all 4 harvests, CR9 and hybrid CR9×CR3 show an aromatic profile dominated by *Z,E*-nepetalactone at the 3^rd^ and 4^th^ successive harvests and, especially in CR9×CR3, essential oils dominated by sesquiterpenes at the 1^st^ and 2^nd^ harvests. For CR9 and CR9×CR3, rosmarinic acid and luteolin diglucuronide were at the highest contents at the 1^st^ and 2^nd^ harvest, while for CR3 the peak occurred at the 3^rd^ successive harvest. Apigenin was only detected in cultivar CR3 from 1^st^ harvest. Despite some variations due to genotype-specific responses, a trend can be observed with a higher accumulation at the 3^rd^ successive harvests of caffeic acid, luteolin, luteolin glucoside, luteolin glucuronide, and apigenin glucoside, glucuronide and diglucuronide. Future studies are necessary to unfold the interactions of these compounds with plant development and productivity. The novel catnip genotypes CR3, CR9 and CR9×CR3 show remarkable potential as sources of multiple natural compounds employed in insect repellent formulations and other industries.

## Data availability statement

The raw data supporting the conclusions of this article will be made available by the authors, without undue reservation.

## Author contributions

EG and JS: conceptualization. EG, HP, HRJ, WL, JS and BY: methodology. EG: Data analysis. EG and HP: original draft preparation. WL, BY, HRJ, QW and JS: review and editing. JS and QW: supervision and funding acquisition. All authors contributed to the article and approved the submitted version.
